# Assessment of Residual Stresses in Laser Powder Bed Fusion Manufactured IN 625

**DOI:** 10.3390/ma17020413

**Published:** 2024-01-14

**Authors:** Alexandru Paraschiv, Gheorghe Matache, Mihai Vladut

**Affiliations:** 1 Special Components for Gas Turbines Department, Romanian Research and Development Institute for Gas Turbines COMOTI, 220D Iuliu Maniu, 061126 Bucharest, Romania; gheorghe.matache@comoti.ro (G.M.);; 2 Section IX-Materials Science and Engineering, Technical Sciences Academy of Romania, 26, Dacia Blvd., 030167 Bucharest, Romania

**Keywords:** PBF-LB/M/IN625, bridge specimens, distortion, residual stresses, Inconel 625

## Abstract

Residual stresses pose significant challenges in the powder bed fusion of metals using a laser (PBF-LB/M), impacting both the dimensional accuracy and mechanical properties. This study quantitatively analyzes deformation and residual stresses in additively manufactured Inconel 625. Investigating both as-built and stress-relieved states with varied scanning strategies (90°, 67°, strip, and 90° chessboard) in PBF-LB/M/IN625, distortion is evaluated using the bridge curvature method. Quantitative measurements are obtained through 3D laser surface scanning on pairs of bridge specimens—one measured before and after detachment from the build plate, and the other undergoing stress-relieving heat treatment at 870 °C for 1 h. The findings reveal that, among as-built specimens, the 90° and 90° strip strategies induce the least distortion, followed by the 67° and chessboard 90° strategies. Furthermore, stress-relief treatment significantly reduces residual stress levels. After post-treatment, the deformation in X-axis samples with 90° and 90° strip strategies decreases by 39% and 42%. In contrast, the samples with the 67° and 90° checkerboard strategies exhibit more pronounced reductions of 44% and 63%, respectively. These quantitative results contribute useful insights for optimizing PBF-LB/M/IN625 processes in additive manufacturing.

## 1. Introduction

The powder bed fusion of metals (PBF-LB/M), also known as selective laser melting (SLM), is an emerging technology that uses a laser beam to selectively melt metal powder particles layer by layer, with many applications in the aerospace, medical, and automotive fields [1,2,3,4,5,6]. Thermal residual stresses are a primary concern in the PBF-LB/M process, given the high temperature gradients and rapid cooling rates associated with it [1,4,7]. Residual stresses in PBF-LB/M or direct energy deposition (DED) techniques are typically higher than in electron beam melting (EBM) due to the cooling rate [1]. Generally, rapid cooling rates in the range of 10^3^ K/s to 10^8^ K/s occurring in the PBF-LB/M process [8,9] lead to material shrinkage and distort the geometry of parts [1,6], impacting both dimensional accuracy and mechanical properties [1,10,11,12]. Numerous studies have explored the formation of residual stresses in materials produced through PBF-LB/M manufacturing. These studies examine the impact on the material of various factors, including process parameters [13,14]—with a specific focus on scanning strategies [5,7,15,16,17]—as well as material properties, heat treatments [11,15,18], and the geometry of additively manufactured parts (AMed) [5]. Other studies have approached this topic by considering a combination of factors. For instance, Mishurova et al. [9] identified a strong correlation between part geometry and factors such as volumetric energy density, heat treatment, and the removal of parts from the base plate in a PBF-LB/M process.

The primary and widely adopted approach to reduce residual stresses in AM parts, performed before detaching them from the baseplate, involves stress relief heat treatment [11,18,19]. Other stress reduction techniques used by various authors considered the preheating the chamber and build plate [5,7,9], decreasing the energy density [6,11,16,17] or changing the scan vector length [5,20]. For instance, decreasing the scan vectors from 20 mm to 2.5 mm has been demonstrated to halve distortion [5]. A higher laser energy density during manufacturing has been associated with a reduction in residual stress [9]. Additionally, preheating the build platform to 180 °C can lead to a 10% decrease in deformation for parts produced through PBF-LB/M [21].

However, it is commonly observed that the scanning direction tends to exhibit the highest reported residual stresses in the PBF-LB/M process [2] and extensive studies have been conducted to explore the impact of scanning strategies on residual stress [20,22,23,24,25]. Thermo-mechanical finite-element models of residual stress have been documented by Parry et al. [20] who analyzed the interaction between temperature history and mechanical response, as well as the impact of the laser scan strategy on stress distribution. Wu et al. [23] investigated the impact of scanning island size and rotation between the island and the wall on residual stresses induced in 316L stainless steel, showing a decrease in residual tensile stress when reducing the island size from 5 × 5 mm to 3 × 3 mm. Compensating for workpiece geometry, as proposed by Afazov et al. [24], is another approach to reduce distortion in PBF-LB/M. Additionally, rotating the scanning direction between consecutive layers is a typical procedure that results in a greater isotropic stress distribution [25,26].

However, understanding their effects and taking appropriate measures to minimize distortions is essential when designing, validating, and certifying AMed parts. The techniques for measuring distortion are classified into non-destructive, destructive, and semi-destructive methods [1]. Common techniques include the hole drilling method [27], slitting method [27], the contour method [28], and diffraction-based methods [29]. Notably, the latter provides information limited to the outer surface layers.

One of the most common techniques used to study residual stress in PBF-LB/M is the bridge curvature method (BCM) which is a technique for measuring the deflection (curl-up angle) caused by residual stress in bridge specimens [1,5,11,14,30,31,32] or cantilever specimens [5,11,15]. The bridge curvature method has become a widely used technique in the additive manufacturing community due to its sensitivity, simplicity, non-destructive nature, and applicability to various materials [3,21]. Its quantitative nature makes it a valuable tool for researchers and industry operators looking to optimize PBF-LB/M processes and improve the overall quality of additively manufactured parts.

Le Roux et al. [4] evaluated the accuracy of the bridge curvature method (BCM) through a statistical analysis using repeatability tests and found that the results obtained from surface topographies offer greater robustness and repeatability compared to isolated profile-based distortion measurements.

Kruth et al. [21] utilized the BCM to evaluate and qualitatively compare the influence of various laser scan patterns and parameters on the residual stress in Ti6Al4V bridges produced by PBF-LB/M. They found that thermal stresses can be diminished by the optimal selection of scan orientation, employing short scan vectors, preheating of the base plate, and stress-relief heat treatment.

In this study, the impact of various scanning strategies (90°, 67°, 90° strip, and 90° chessboard) in PBF-LB/M manufacturing on the distortion of Inconel 625 components, both in the as-built and stress-relieved states, was investigated using the bridge curvature method.

## 2. Materials and Methods

**Figure 1:**
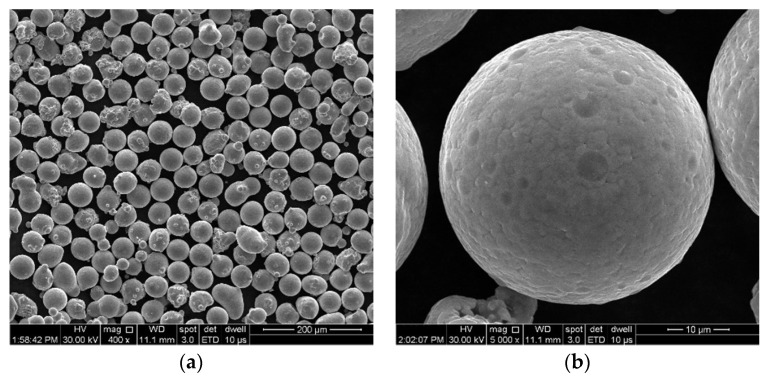
SEM images of IN 625 powder: (**a**) image at 400×; (**b**) image at 5000×.

For this study, bridge-type test pieces (Figure 1) were manufactured using a Lasertec 30 SLM (DMG Mori, Bielefeld, Germany) and vacuum gas-atomized IN 625 metal particles (supplied by LPW Technology Ltd., Runcorn, UK). The raw material’s morphology is depicted in the scanning electron microscopy (SEM) images in Figure 1a,b, acquired using the FEI F50 Inspect microscope (FEI Company, Brno, Czech Republic), and its chemical composition is presented in Table 1. According to the manufacturer’s batch test certificate, the metal powder consists of spherical particles with sizes ranging from 15 to 45 µm, and it has a particle size distribution of D10 = 20 ± 2 µm, D50 = 30 ± 5 µm, and D90 = 45 ± 5 µm.

**Figure 2:**
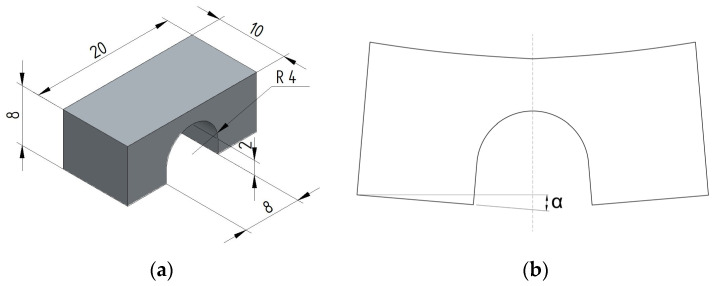
Bridge-type test piece used for distortion measurements: (**a**) dimensions in millimetres of the bridge specimen on the build plate and (**b**) after separation from the build plate.

To assess part distortion, bridge-type test pieces, with dimensions in millimetres depicted in Figure 2, were manufactured via PBF-LB/M and subsequently measured after removal from the build plate.

Except for the scanning strategy which was varied, the following process parameters (standard parameters) were used for the manufacture of the bridge samples: 250 W laser power, 750 mm/s laser speed, 40 µm layer thickness, and 0.11 mm hatch distance. Also, the samples were built with no contour strategy. Prior studies conducted by the authors [33,34,35,36] demonstrated that these parameters are optimal in terms of the density, microstructure, and mechanical properties of the IN 625 alloy manufactured using the same Lasertec 30 SLM machine. This approach was adopted to facilitate a more precise understanding of the isolated impact of scanning strategies on the results, independent of other variables.

**Figure 3:**
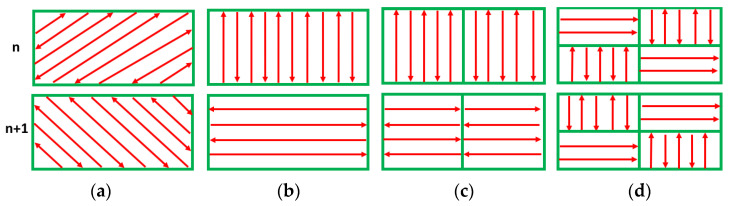
Bi-directional scanning strategies used for manufacturing the IN 625 bridge samples. (**a**) 67°, (**b**) 90°, (**c**) 90° strip, (**d**) 90° chessboard.

The bridge samples were manufactured with no contour using the following process parameters: 250 W laser power, 750 mm/s laser speed, 40 µm layer thickness, and 0.11 mm hatch distance. These bridge pieces were built using various scanning strategies between adjacent layers, including 90°, 67°, 90° strip, and 90° chessboard, as illustrated in Figure 3.

**Figure 4:**
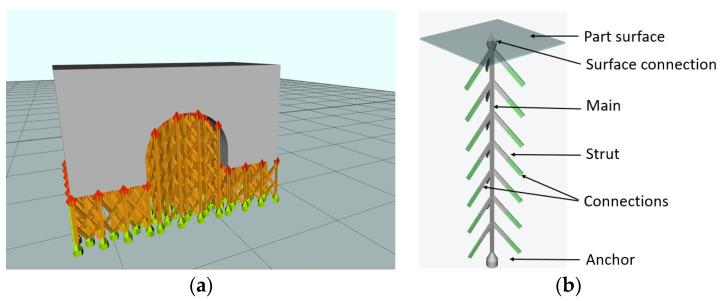
Bridge-type test piece used for distortion measurements: (**a**) bridge with supporting structures (RDesigner capture) and (**b**) geometry of the structure support.

The same process parameters for the support structures, as illustrated and presented in Figure 4a,b and Table 2, respectively, were used to manufacture all specimens ensuring secure adherence to the build plate. The fine-tuning of these process parameters started from the predefined process parameters recommended by the equipment manufacturer. This approach was chosen to maintain a reliable and standardized foundation for the manufacturing process.

Pairs of test pieces for each scanning strategy were manufactured parallel to the X- and Y-axes. Parts distortion as a function of different scanning strategies was investigated using 3D laser surface scanning to analyze the change in the sample surface profile before and after cutting from the build plate. All test pieces were initially measured on the build plate. One test piece from each pair was cut to be analyzed in its as-built condition, while the other was remained on the build plate to undergo stress-relieving heat treatment. Heat treatment in air was conducted on bridge-type test pieces using an electrical Nabertherm LH 30/14 chamber furnace (Nabertherm GmbH, Lilienthal, Germany). The stress-relieving heat treatment involved heating from room temperature to 870 °C, holding for 1 h, and subsequent cooling in air to room temperature. The heat treatment conditions applied to additively manufactured (AMed) IN 625 were adapted; initially, the standard heat treatment protocol for conventionally manufactured IN 625 was followed, using the same stress-relief temperature.

The 3D laser surface scanning was performed using a “NIKON Altera 10.10.8” coordinate measuring machine (LK Metrology Ltd., Derby, UK) equipped with a non-contact NIKON LC15Dx laser scanning probe (LK Metrology Ltd., Derby, UK), with an accuracy of 1.9 µm. The machine uses software FOCUS 2019 (R2, Nikon Metrology NV, Leuven, Belgium) for post-processing and analysis of numerically extracted data using dedicated software. The surface texture was globally evaluated with an analysis allowance set to 5%. The FOCUS software can analyze each section and numerically extract and export data. The exported data were used to generate diagrams and quantitatively assess sample distortion. To ensure credibility and eliminate potential sources of variation, a standardized procedure was applied to establish the zero point for all samples during scanning and data analysis. This approach ensured that any error, if present, would be consistently applied across all measurements.

The specimens were manufactured using a cross-type support structure with the geometry presented in Figure 4, along with the dimensions and process parameters shown in Table 2. The samples were built on a preheated build plate (80 °C), with argon flow maintained to ensure a 0.2% oxygen content within the building chamber.

## 3. Results

**Figure 5:**
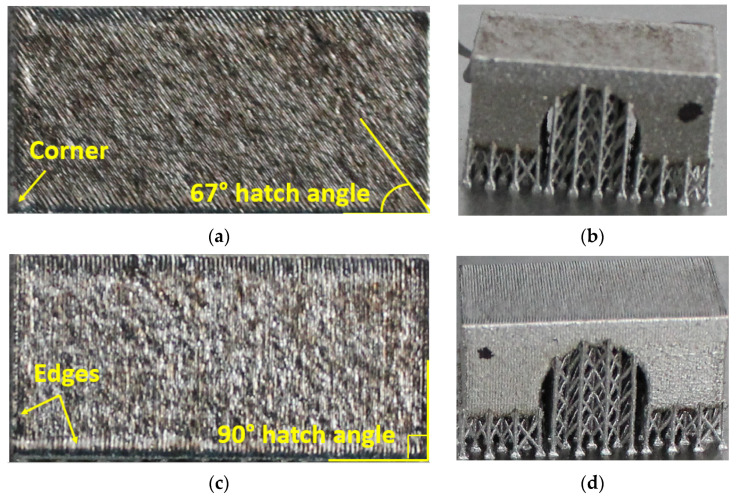

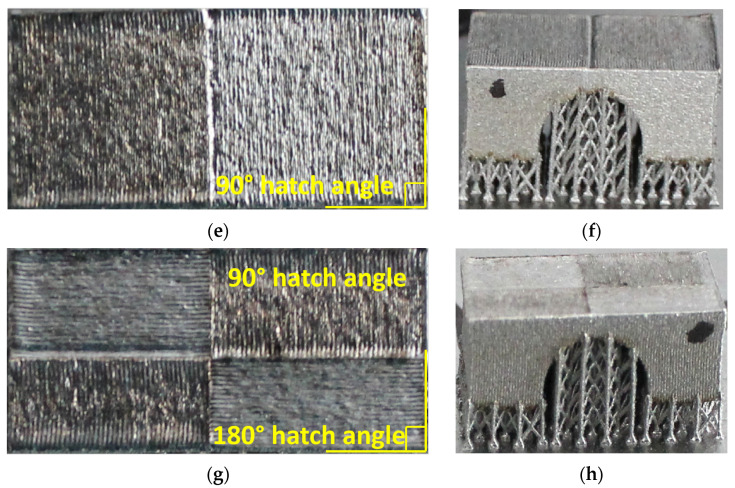
Macroscopic top and lateral views of the bridge pieces built with different different scanning strategies: (**a**,**b**) 67°, (**c**,**d**) 90°, (**e**,**f**) 90° strip, and (**g**,**h**) 90° chessboard.

Comparative analyses were conducted on bridge samples built using scanning strategies of 90°, 67°, 90° strip, and 90° chessboard. Figure 5 depicts macrographs of the top and lateral surfaces of the samples before removal from the build plate.

**Figure 6:**
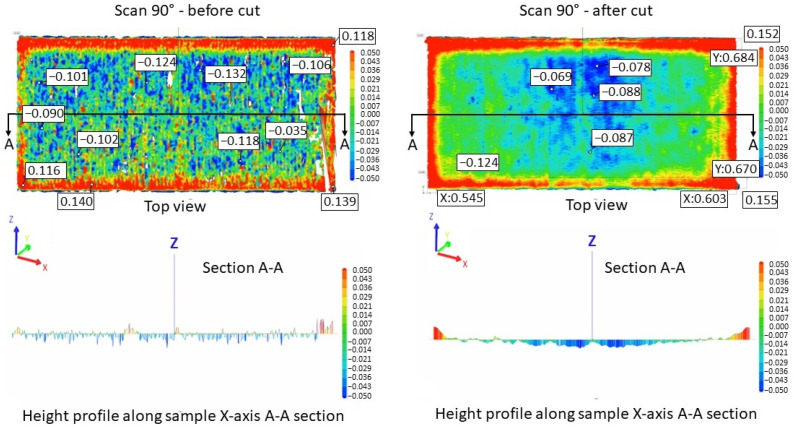
Height profiles of samples built using a 90° scanning strategy on the building plate before cutting (**left**) and after cutting from the building plate (**right**).

**Figure 7:**
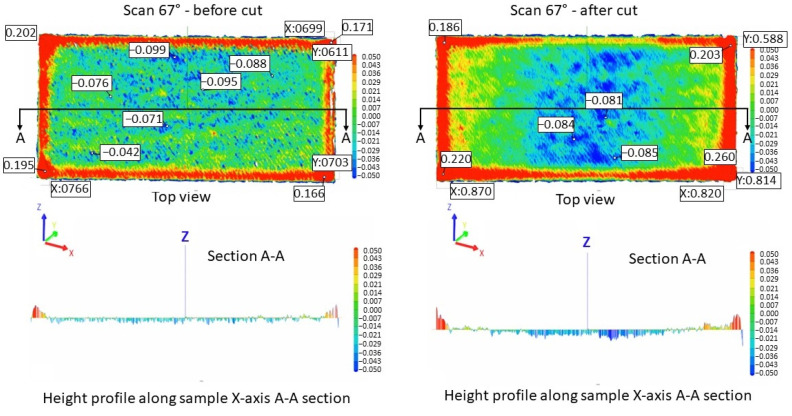
Height profiles of samples built using a 67° scanning strategy on the building plate before cutting (**left**) and after cutting from the building plate (**right**).

**Figure 8:**
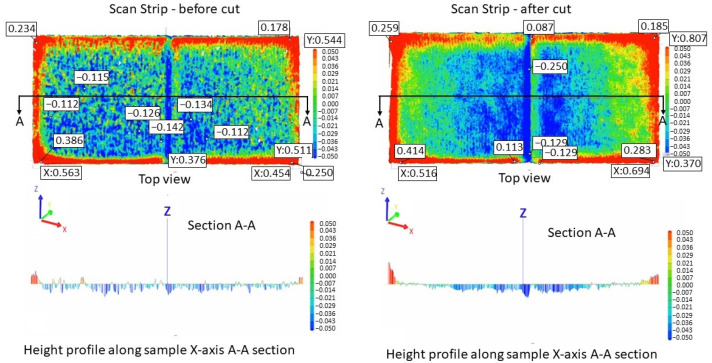
Height profiles of samples built using a 90° strip scanning strategy on the building plate before cutting (**left**) and after cutting from the building plate (**right**).

**Figure 9:**
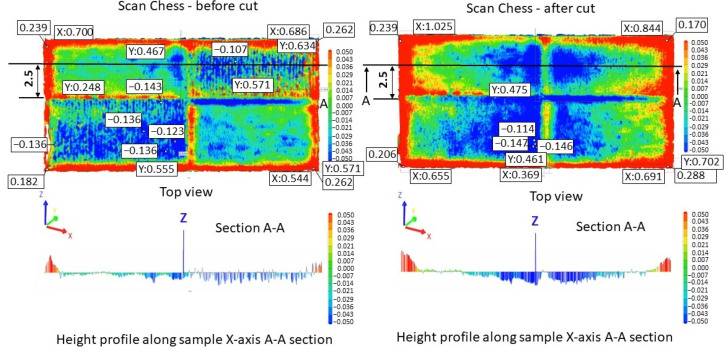
Height profiles of samples built using a 90° chessboard scanning strategy on the building plate before cutting (**left**) and after cutting from the building plate (**right**).

Figure 5 illustrates the surface texture of the top and lateral surface samples resulting from scanning strategies of 90°, 67°, 90° strip, and 90° chessboard, including melting tracks, contour tracks, and elevated ridges and corners. The corresponding 3D top surfaces of the bridge samples were obtained via 3D laser surface scanning. Figure 6, Figure 7, Figure 8 and Figure 9 present the 3D top surfaces and height profiles before and after cutting from the build plate of test pieces built parallel to the X-axis of the machine.

**Figure 10:**
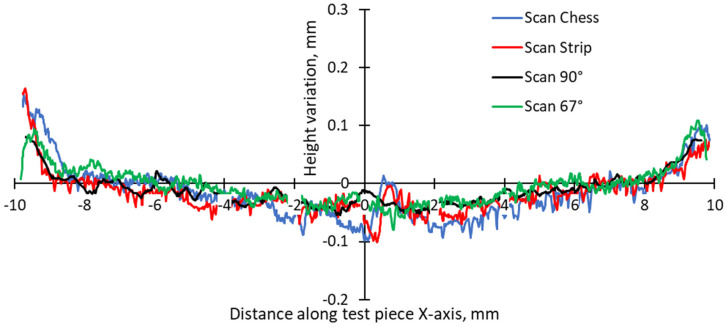
Height profiles along test pieces’ sections.

The 3D top surfaces of the bridge samples exhibit particularities depending on the selected scanning strategy. During rapid solidification, the elevated ridges become more prominent, especially in the cases of samples built with the 67° and chessboard scanning strategies. This adverse effect is primarily attributed to compressive stress around the corners and the thermal warping effect [37,38], which can lead to significant distortions in additively manufactured components [36]. As seen in Figure 6, Figure 7, Figure 8 and Figure 9, the distortions of bridge pieces become more pronounced after the removal from the base plate (after cut). This behavior was also found by Robinson et al. [25] in their study and is attributed to the release of residual stresses resulting from shrinkage and bending deformation [37]. To quantify the distortion of the parts after cutting from the build plate, height profiles were drawn along the test piece’s X-axis sections. Figure 10 presents the overlapped height profiles for the test pieces built parallel to the X-axis of the machine using different scanning strategies.

**Figure 11:**
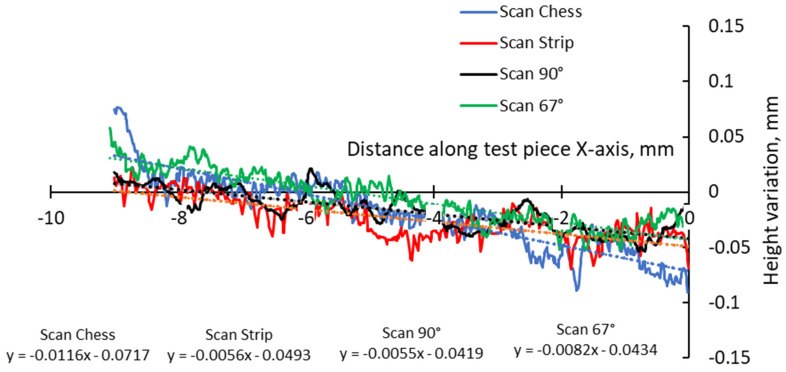
Comparison of height profiles of test pieces built with different scanning strategies and associated slopes.

The distortion of the as-built test pieces was assessed by calculating the slopes of the curves in the two halves of the sample relative to the symmetry axis. These slopes can be easily converted to angles. Figure 11 presents the calculated slopes for different scanning strategies. It can be noticed that the chessboard scanning strategy generates higher distortion than the 90° strip and 90°. The latter two are also very close.

**Figure 12:**
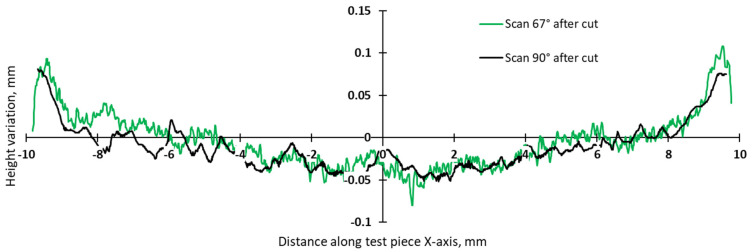
Height profiles along the X-axis of test pieces built using 67° and 90° scanning.

A more detailed analysis was conducted to specifically assess the effects of hatch angles, particularly at 90° and 67°. Figure 12 presents the complete height profile along the X-axis of the test pieces.

This focused approach was chosen to mitigate the influence of other factors, such as the scanning vector length, the arrangement of scanning islands, and thermal gradients. These factors are directly influenced by the pattern, where the scan could be an entire layer, separated islands, or a stripe in the chessboard. Although 90°, 45°, and 67° are commonly used hatch angles for bi-directional scanning, a previous study on top surface deformation of AMed IN 625 demonstrated that the differences in terms of edge width and height between 90° and 67° angles are relatively close, while the hatch angle of 45° induces the highest deformations [36].

**Figure 13:**
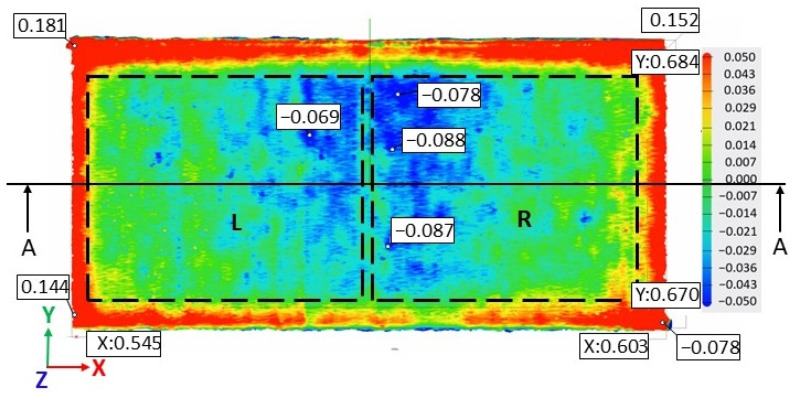
Schematic representation of section A-A along the X-axis of test pieces.

The slope calculation for the two scanning strategies was performed in section A-A along the X-axis in the two halves, denoted as left (L) and right (R), of the test pieces as depicted in Figure 13.

**Figure 14:**
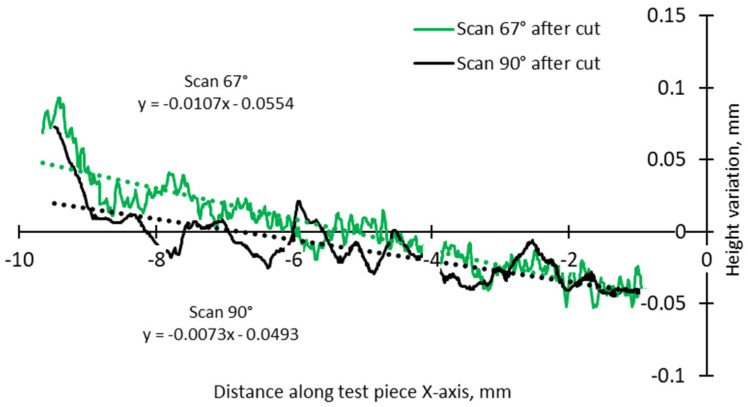
Comparison of height profiles of test pieces built with 67° and 90° scanning strategies and associated slopes (half left—L).

**Figure 15:**
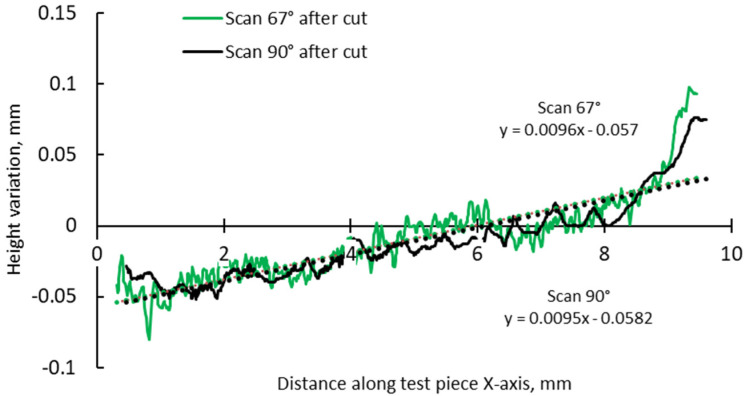
Comparison of height profiles of test pieces built with 67° and 90° scanning strategies and associated slopes (half right—R).

The slope calculation in the two halves of the test pieces shows that the distortion induced by the 90° scanning strategy is somewhat lower than even in the case of 67° (Figure 14 and Figure 15).

Based on the results obtained on the as-built test pieces manufactured in the parallel direction with the X-axis of the machine, the smallest distortions are induced by the 90° and 90° strip strategies. These are followed, in order, by the 67° and chessboard 90° scanning strategies.

During the PBF-LB/M process, residual stresses accumulate in the part, and when it is removed from the plate, a redistribution of stresses and deformations occurs. Typically, the bottom of an PBF-LB/M part is subjected to compression stress [5], while the top experiences tensile stress [17]. During cooling, a recently added layer tends to shrink, constrained by the underlying layer, leading to tensile stress in the new layer and compressive stress in the lower layer [1].

**Figure 16:**
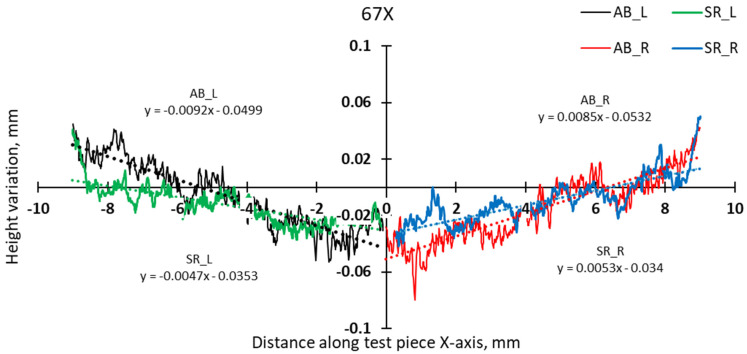
Comparison of height profiles of test pieces built in the X direction with the 67° scanning strategy and associated slopes (half left—L, half right—R).

**Figure 17:**
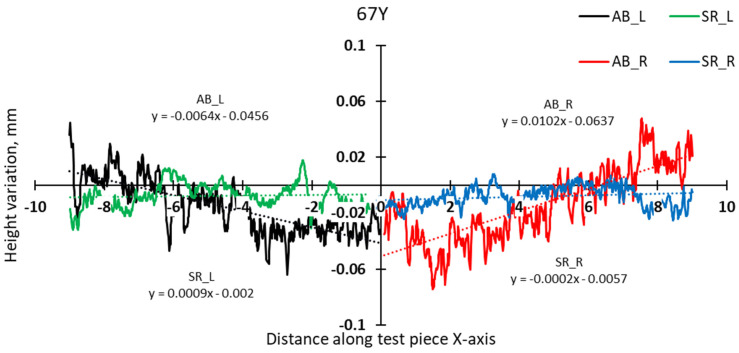
Comparison of height profiles of test pieces built in the Y direction with the 67° scanning strategy and associated slopes (half left—L, half right—R).

**Figure 18:**
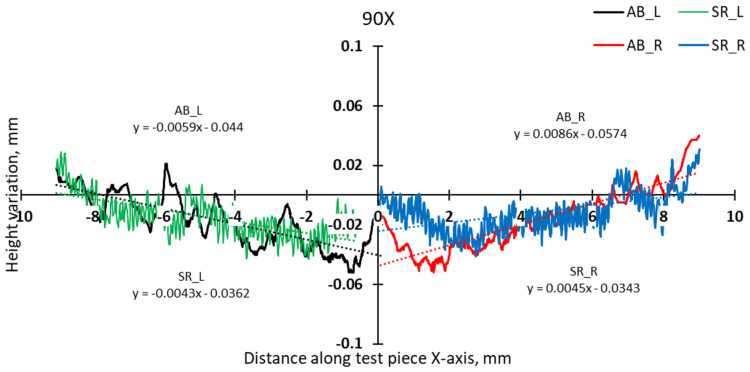
Comparison of height profiles of test pieces built in the X direction with the 90° scanning strategy and associated slopes (half left—L, half right—R).

**Figure 19:**
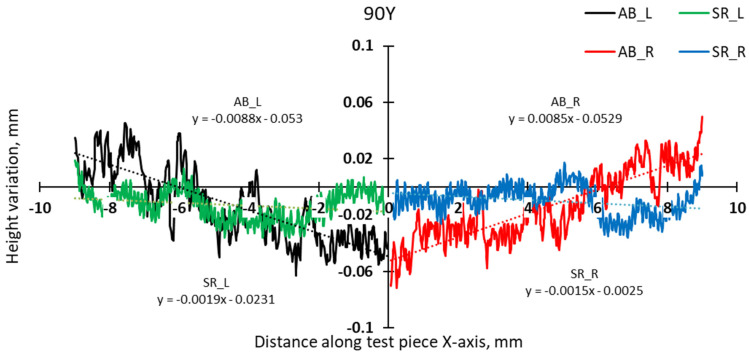
Comparison of height profiles of test pieces built in the Y direction with the 90° scanning strategy and associated slopes (half left—L, half right—R).

The comparative analysis of sample deformations before and after heat treatment showed that a stress-relief treatment conducted at 870 °C for 1 h is effective in reducing the residual stress level. Figure 16, Figure 17, Figure 18 and Figure 19 present the profiles of the bridge samples after cutting from the build plate in as-built (AB) and stress-relieved (SR) conditions.

**Figure 20:**
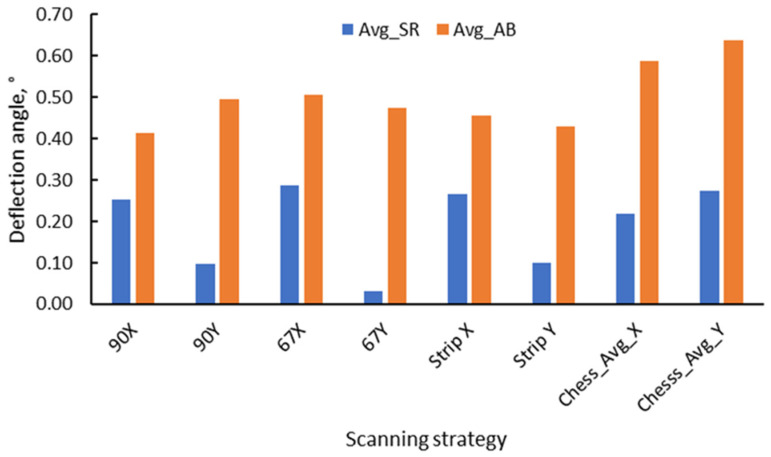
Average deflection angle of bridge test pieces built with different scanning strategies after cutting from the build plate.

Based on the left and right slopes of the test pieces’ height variation, deflection angles of the samples were calculated. Figure 20 summarizes the comparison of the average deflection angles (left and right) for the different scanning strategies used for both as-built and stress-relieved bridge samples.

Quantitative analysis of distortion, assessed through slope calculations and angle conversions, offers compelling insights on bridge sample deformation and, consequently, residual stresses, exhibited variations based on the sample state and scanning strategy. As shown in Figure 20, there is a significant decrease in the internal stress level of the samples after heat treatment. Regarding the effects of the X- and Y-axis manufacturing, with the exception of the chessboard scanning strategy, the results demonstrated that the stress-relieved bridge samples built parallel to the Y-axis exhibited lower stress levels compared to X-axis.

## 4. Discussion

This study investigates the influence of scanning strategies on distortion in Inconel 625 components manufactured by PBF-LB/M. A comprehensive comparative analysis of scanning strategies—90°, 67°, 90° strip, and 90° chessboard—provides valuable insights into the complex dynamics governing part distortion and residual stresses. Recent studies have further assessed residual stresses and distortions in additively manufactured materials, primarily focusing on metallic alloys such as 316L stainless steel [25,39], Inconel 718 [40], and Ti6Al4V [9,41]. However, it is noteworthy to recognize a limitation in AM technology concerning result repeatability, especially when using the same material but with different machines or parameters.

Macrographs and 3D laser surface scanning revealed distinctive surface textures resulting from various scanning strategies. During rapid solidification, the elevated ridges increased, especially in samples constructed using the 67° and chessboard scanning strategies, which challenges prevailing findings about the distortion-reducing efficacy of the chessboard strategy [10]. This behavior is attributed to compressive stress and the thermal warping effect, accentuated upon detachment from the base plate. The proposed temperature gradient mechanism (TGM) model by Mercelis et al. [42] aligns with our observation emphasizing the localized effects of heating and cooling cycles on compressive and tensile residual stresses in the heat-affected zone. Mishurova et al. [9] measured the distortion angles of the bridge-shaped specimens and lattice strain using microscopy and energy-dispersive diffraction methods. They observed that laser energy, scanning speeds, and scan patterns significantly influence solidification and heat distribution, thereby inducing residual stress in PBF-LB/M parts. These findings are consistent with previous research on IN 625 manufactured via the PBF-LB/M technique. In a previous study [36], a parallel investigation was conducted on the top surface deformation of IN 625 cubes manufactured using similar manufacturing and characterization conditions, process parameters, and identical equipment. This study explored various factors, including the presence or absence of a contour strategy, different hatch angle rotations, reductions in volumetric energy density (VED) for the last layer(s) of parts, and remelting the last layer once or several times. The findings from this study revealed that IN 625 samples built with hatch angle rotations of 45° and 67° had 72% and 20% higher deformations, respectively, compared to those built with a rotation angle of 90° between successive layers. Notably, a 90° hatch angle rotation induces a checkerboard pattern due to epitaxial solidification, resulting in reduced deformation in PBF-LB/M components compared to the 45° and 67° hatch angle rotations, leading to a hexagonal grain orientation [36]. Introducing the contour strategy, the corner height of samples built with hatch angles of 45°, 67°, or 90° were reduced by 17%, 31%, and 19%, respectively, compared to the same samples without contour. Moreover, the same study demonstrated that reducing the volumetric energy density (by lowering the laser power from 250 W to 150 W and increasing the scanning speed from 0.75 m/s to 0.9 m/s), employing the contour strategy, or applying the laser surface remelting technique significantly diminishes the distortion of parts manufactured by PBF-LB/M [36]. This establishes a clear correlation between these variables and the deformations observed in IN 625 parts produced through PBF-LB/M. Nevertheless, in the current study, the process parameters were selected for the manufacturing of bridge-type test pieces based on their excellent results in terms of density, microstructure, and mechanical properties, as demonstrated in previous studies [33,34,35,36]. This approach was intentionally adopted to facilitate a more precise understanding of the isolated impact of scanning strategies on results, independent of other variables.

To determine the impact of stress-relieving heat treatment in reducing residual stress, careful consideration must be given to the selection of temperature and duration, particularly about the microstructure and mechanical properties of additively manufactured parts. The choice of temperature and conditions for the heat treatment was guided by previous studies conducted by the authors [33,34,35] on additively manufactured IN 625 alloy, specifically examining its effects on microstructure and properties. However, a stress-relief heat treatment typically ranges from 650 to 870 °C [43], and the selection of 870 °C for IN 625 aligns with industry standards [44]. The findings from these studies support the conclusion that the selected treatment is also suitable for stress relief in AMed IN625.

Considering that heat treatment is known to reduce residual tensile stresses [1], this study aimed to anticipate and quantify the effects of this treatment on the AMed IN625. In all cases, there is a significant reduction in the internal stress level of the samples after heat treatment, which leads to a decrease in residual stresses. Specifically, in the case of samples built along the X-axis post heat treatment and detachment from the build plate, the deformation in samples constructed with 90° and 90° strip strategies decreased by 39% and 42%, respectively. Conversely, samples built with 67° and 90° chessboard scanning strategies experienced a more pronounced decrease, with reductions of 44% and 63%, respectively. This behavior aligns with Li et al. [39] which found a significant reduction in tensile residual stress (53.7% decrease to 356.29 MPa) in 316L stainless steel fabricated using directed laser deposition compared to untreated samples. Wang et al. [40] observed a reduction in the maximum absolute residual stress from 378 MPa to 321 MPa in Inconel 718 fabricated using the PBF-LB/M process after applying stress-relieving heat treatment. In the case of Ti6Al4V bridges, the reduction in residual stress had a substantial impact, resulting in an 80% decrease in the measured angle of deformation after the application of a specific heat treatment [21].

In X- and Y-axis manufacturing, stress-relieved bridge samples along the Y-axis generally exhibit lower stress levels than the X-axis. For as-built test pieces in the X-axis direction, minimal distortions occur with 90° and 90° strip strategies, followed by 67° and chessboard 90° strategies. This directional influence is attributed to the predominant alignment of residual stress with the scanning direction [41], further supported by the scan length variation between the X- and Y-axes. These findings align with the results obtained by Robinson et al. [25] who assessed the impact of various scan techniques on residual stress in PBF-LB/M. They found that specimens built on the X-axis exhibited greater stress than those built on the Y-axis. This is attributed to the primary residual stress being generated parallel to the direction of scanning. Additionally, their findings indicated contradictory results in studies on the influence of the scan direction on residual stress. Initial investigations suggested that the predominant residual stress was perpendicular to the scan direction. However, recent studies have largely contradicted this, asserting that the highest stress aligns parallel to the scan vectors [25].

Kruth et al. [21] found that the angle deformation decreases as the vector length decreases with more pronounced effects at shorter lengths. A significant improvement of a 13% reduction in curling angle is observed with vector lengths of 2 mm, compared to the reference part which has a vector length of 20 mm. Extended scan vectors facilitate cooling of the previously scanned region as the laser beam covers a considerable distance. This leads to significant temperature differentials between the scanned area and the new scan line, generating higher thermal stresses [45]. Conversely, when the scan tracks are very short, scanning strategies geometrically impact on the accumulation of residual stress, as demonstrated by Parry et al. [20].

This discussion does not entirely elucidate the complicated relationships between scanning strategies, process parameters and residual stresses, aiming at this stage only to pave the way for future research. Future investigations could focus on optimizing scanning strategies, considering the impact of scan vector length for different scan geometries and sample sizes, as well as material-specific responses and manufacturing orientations, with the aim of improving the reproducibility and reliability of additive manufacturing processes. This exploration should extend to various metallic materials and other machines utilizing PBF-LB/M technology or other technologies within the metallic additive manufacturing domain.

In metallic additive manufacturing, the repeatability of results is a sensitive topic, particularly when utilizing the same material with different machines or parameters. The evident variability in results across different machines and parameter settings highlights the need for careful consideration. By combining the results obtained in the present study with those from a previous study [36] on internal stresses and deformations in IN 625 samples, future research can be strategically designed. One potential direction involves exploring whether similar distortion trends are observed in other samples with varying sizes and geometries. Another intriguing approach is to assess residual stress in AMed IN 625 by examining distortions on bridge specimens fabricated along the X-Y-Z axes, as well as tilted at 45° both horizontally and vertically, using the contour and 90° scanning strategy. This includes implementing stress-relief heat treatment and applying lower VED energy for the last layer(s).

## 5. Conclusions

The careful selection of scanning strategy emerges as a pivotal factor influencing distortion in components manufactured using PBF-LB/M. This study investigated the distortion of Inconel 625 components in both as-built and stress-relieved states, focusing on the influence of different scanning strategies (90°, 67°, strip, and 90° chessboard).

Among the as-built specimens, the 90° and 90° strip scanning strategies induced the least distortion, followed by the 67° and chessboard 90° strategies. Notably, this challenges the common assumption that the chessboard scanning strategy reduces distortion.

Furthermore, stress-relieving heat treatment at 870 °C for 1 h proved effective in reducing residual stress levels in PBF-LB/M/IN625 components, contributing to enhanced part quality and mechanical properties.

The orientation of part manufacturing relative to the X- and Y-axes also influenced stress levels, with samples built parallel to the Y-axis exhibiting lower stress levels compared to those built parallel to the X-axis. This outcome is attributed to the direction of residual stress aligning with the scanning direction.

The findings from this study on residual stresses in the additive manufacturing process align with previous research on similar materials and techniques. Overall, understanding and managing residual stresses in PBF-LB/M/IN625 processes are critical for achieving a better dimensional accuracy and mechanical performance in additively manufactured parts.

The choice of scanning strategy significantly impacts distortion levels, and stress-relieving heat treatment proves to be an effective method for improving part quality and dimensional accuracy. The findings obtained in this study not only contribute to the current understanding of additive manufacturing processes but also pave the way for future investigations and refinements in optimizing additively manufactured IN 625 components.

## Figures and Tables

**Table 1:** Chemical composition of IN 625 powder.

Element (%wt.)	Al	C	Co	Cr	Fe	Mn	Mo	Nb	Si	Ti	Ni
Specification	<0.4	<0.1	<1.0	20–23	3–5	<0.5	8–10	3.15–4.15	<0.5	<0.4	Bal.
Actual composition	0.06	0.02	0.1	20.7	4.1	0.01	8.9	3.77	0.01	0.07	Bal.

**Table 2:** Process parameters of structure supports used for manufacturing of the bridge samples.

Surface Selection	Values/Types	Connections	Values/Types
Upper Angle [degrees]	35	Min Count	3
Lower Angle [degrees]	0	Max Count	3
Surface Angle [degrees]	45	Min Distance [mm]	1
Boundary Offset [mm]	0.02	Max Distance [mm]	2
Surface Offset [mm]	0.4	Start Distance [mm]	0.5
Support Type	Boundary	Z-Distance [mm]	2
Boundary Distance [mm]	2	Connecting Angle [degrees]	45
Offset Distance [mm]	0.5	Surface Connection	
Anchor		Geometry [mm]	Cross
Geometry	Circle	Size [mm]	0.8
Size [mm]	0.8	Height [mm]	0.6
Height [mm]	0.5	Shrinking	Activated
Strut		Main	
Geometry	Line	Geometry	Cross
Size [mm]	0.3	Size [mm]	0.3

## Data Availability

Data are contained within the article.
